# Editorial to “Acute occlusion of the left main coronary artery following impedance rise after high‐frequency catheter ablation”: Prepare for a disastrous matter in the EP laboratory

**DOI:** 10.1002/joa3.13132

**Published:** 2024-08-11

**Authors:** Satoshi Higa

**Affiliations:** ^1^ Cardiac Electrophysiology and Pacing Laboratory, Division of Cardiovascular Medicine Makiminato Central Hospital Okinawa Japan

In this issue of the *Journal of Arrhythmia*, Koyama et al.^1^ reported a case of frequent premature ventricular contractions complicated with a left main coronary artery (LMCA) occlusion post‐ablation in the left coronary cusp (LCC). Although they emergently performed angioplasty and implanted a drug‐eluting stent, coronary angiography showed a 99% in‐stent acute stenosis requiring repeat balloon dilatations. Intravascular ultrasound (IVUS) revealed intimal thickening and tissue protrusion within the stent. Finally, the in‐stent restenosis completely resolved after additional balloon dilatations. The patient was successfully weaned from assisted circulation and was discharged on postoperative Day 7. During a 6‐year long‐term follow‐up, the patient remained free of stent restenosis.

Current guidelines provide enough evidence of highly successful outcomes with overall cure rates of catheter ablation of idiopathic ventricular arrhythmias (VAs) and propose as a first‐line therapy. However, successful ablation cannot be obtained in some populations due to anatomic limitations. For this particular reason, one of the most challenging issues that physicians may encounter in the EP laboratory is the approach to VAs originating from the left ventricular summit (LVS). Highly variable complex anatomies between the LVS and neighboring structures emphasizes the importance of a detailed characterization of the individual anatomy of this region and the use of 3D‐anatomical reconstructions using image integration of ICE (intracardiac echocardiography) or a computed tomography for precise and safe mapping and ablation procedures. Although, the LVS can be accessed directly via an epicardial approach, the approach to this superior region usually is very limited due to the close proximity to the LMCA and thick fat layer. Practically, VAs originating from the epicardial aspect of the LVS can be targeted from the anterior interventricular vein (AIV)/great cardiac vein (GCV). On the other hand, the endocardial to intramural aspect of the LVS can be approached from the LCC, left ventricular outflow tract (LVOT) endocardium, or right ventricular outflow tract (RVOT) septal side. Due to the close proximity of multiple structures and nature of the preferential conduction around this region, the pace mapping method's efficacy for localizing VA origins may be poor. Therefore, activation mapping during spontaneous VAs is mandatory for localizing VA origins. In general, an earlier activation time in the distal GCV or proximal AIV than other sites within the RV/LVOT suggests epicardial LVS VAs. Regarding the ECG characteristics of the author's case, the previous algorithm using the aVL/aVR Q‐wave ratio for LVS VAs supports a GCV/AIV region.^2^ Furthermore, finding an earlier ventricular activation time preceding the QRS onset and unipolar electrogram morphology with a QS pattern also suggest a GCV/AIV origin. For more accurate localization of VA origins, epicardial LVS mapping using a 0.014‐inch guidewire or 2Fr multiple electrode catheter through the GCV/AIV can be feasible. Ablation procedure problematic issues within the GCV/AIV are the difficulty in advancing ablation catheters to target sites because of relatively smaller vessel sizes, inability to apply an appropriate radiofrequency energy due to high impedances and/or insufficient blood cooling flow, and close proximity to the LMCA. If ablation within the GCV/AIV is not feasible or unsuccessful ablation results, the next mapping/ablation target would be the LCC, LVOT endocardial, or RVOT septal side. In this case, the ventricular activation time and an rS unipolar electrogram morphology suggest that the LCC was not an ideal target. However, the authors attempted to ablate in the LCC because ablating in the AIV failed due to a high impedance. According to a multicenter study, the procedural success rate of the conventional approach is relatively low despite both endocardial and/or epicardial approaches.^3^ To overcome unfavorable outcomes correlated with anatomical limitations, several alternative techniques including ablation using small 5Fr‐ablation catheters, low energy ablation using a guidewire, anatomical bipolar ablation, and focal monopolar pulsed field ablation can be used for eliminating epicardial VAs.

The case report by Koyama et al.^1^ highlights safety concerns when performing ablation in the coronary cusp and the requirement for immediate management of a lethal complications. LMCA occlusions and thromboembolisms can cause severe an acute myocardial infarction or in‐hospital death. The authors suggested two potential causes underlying the acute LMCA occlusion in this case. Firstly, radiofrequency energy could induce heat conduction from the left coronary artery ostium and cause subsequent LMCA thermal injury. Secondly, temporary catheter migration into the LMCA and accidental intracoronary radiofrequency applications could induce direct thermal injury causing an LMCA occlusion. Although the authors provided IVUS imaging exhibiting intimal thickening and tissue protrusion within the stent, the precise mechanism of the LMCA's acute occlusion and persistent in‐stent restenosis that required repeated balloon inflations remains unclear. In a previous review article, Castaño et al.^4^ summarized three mechanisms of radiofrequency energy‐induced acute coronary artery occlusions including coronary spasms by heat‐induced nervus terminalis modulation, direct vessel trauma by collagen shrinkage and subsequent vessel narrowing, and thromboembolisms. Interestingly, radiofrequency energy delivered adjacent and parallel to vessels results in lesions formation limited to the intima‐media. However, radiofrequency energy delivered directly and perpendicularly to vessels induces severe intimal hyperplasia and thrombosis formation. Therefore, both the radiofrequency energy's extent and catheter tip orientation with the coronary artery are very important considerations for electrophysiologists.

Catheter ablation in the coronary cusp can be complicated by potentially disastrous outcomes in cases with LMCA involvement. LMCA injury is a rare but potentially life‐threatening complication. A retrospective analysis by Klaudel et al.^5^ demonstrated 22 cases of serious LMCA damage identified between 1987 and 2018. Eighty‐six percent of LMCA trauma cases manifest with dramatic life‐threatening arrhythmias, cardiogenic shock, or severe hypotension. The in‐hospital mortality rate is high (32%) and direct stenting is the most effective strategy. Considering safety issues, visualization of the coronary artery by angiography and/or an ICE should be mandatory during ablation in this area. Physicians also should perform careful impedance monitoring to avoid catheter migration into vessels. Furthermore, adequate sedation of the patient is very important to minimize sudden uncontrolled motions and to enhance catheter stability for avoiding catheter dislodgement.

To ensure emergent management, electrophysiologists need to establish a team‐based cross‐specialty approach with close corporation with highly experienced coronary interventionists and cardiovascular surgeons.

## FUNDING INFORMATION

None.

## CONFLICT OF INTEREST STATEMENT

The author has potential conflicts of interest: S.H. is a consultant to Japan Lifeline, Johnson & Johnson, Boston Scientific, and Medtronic, and received speaker's honoraria from Japan Lifeline, Johnson & Johnson, Boston Scientific, Medtronic, Abbott, and Biotronik, and the Boehringer‐Ingelheim, Bristol‐Myers, Bayer, Pfizer, Daiichi‐Sankyo, Mochida and Ono Pharmaceutical Companies.

## ETHICS STATEMENT

None.

## PATIENT CONSENT STATEMENT

None.

## CLINICAL TRIAL REGISTRATION

None.

## Data Availability

None.
